# Restoring GM1 ganglioside expression ameliorates axonal outgrowth inhibition and cognitive impairments induced by blast traumatic brain injury

**DOI:** 10.1038/srep41269

**Published:** 2017-01-23

**Authors:** Vardit Rubovitch, Yael Zilberstein, Joab Chapman, Shaul Schreiber, Chaim G. Pick

**Affiliations:** 1 Department of Anatomy and Anthropology, Sackler Faculty of Medicine, Tel-Aviv University, Tel-Aviv, 69978,Israel; 2 Sackler cellular & molecular imaging center, Sackler Faculty of Medicine, Tel-Aviv University, Tel-Aviv, 69978, Israel; 3 Department of Neurology, The J. Sagol Neuroscience Center, The Chaim Sheba Medical Center, Tel HaShomer, Ramat Gan, Israel; 4 Department of Neurology, The Sackler School of Medicine, Tel Aviv University, Tel Aviv, Israel; 5 Sagol School of Neuroscience, Tel Aviv University, Tel Aviv, Israel; 6 Department of Psychiatry, Tel Aviv Sourasky Medical Center and Sackler Faculty of Medicine, Tel-Aviv University, Tel-Aviv, 64239, Israel; 7 Department of Psychiatry, The Sackler School of Medicine, Tel Aviv University, Tel Aviv, Israel

## Abstract

Blast induced traumatic brain injury (B-TBI) may cause various degrees of cognitive and behavioral disturbances but the exact brain pathophysiology involved is poorly understood. It was previously suggested that ganglioside alteration on the axon surface as well as axonal regenerating inhibitors (ARIs) such as myelin associated glycoprotein (MAG) were involved in axonal outgrowth inhibition (AOI), leading to brain damage. GM1 ganglioside content in the brain was significantly reduced while GD1 ganglioside was not affected. The axonal regeneration was also reduced as seen by the phosphorylated NF-H expression. Moreover, B-TBI induced a significant elevation in MAG expression in the brains of the injured mice. The blast injured mice exhibited a significant decline in spatial memory as seen by the Y-maze test. In addition, the injured mice showed pronounced damage to the visual memory (as evaluated by the Novel object recognition test). A single low dose of GM1 (2 mg/kg; IP), shortly after the injury, prevented both the cognitive and the cellular changes in the brains of the injured mice. These results enlighten part of the complicated mechanism that underlies the damage induced by B-TBI and may also suggest a potential new treatment strategy for brain injuries.

Traumatic brain injury (TBI) is a major public health concern and the victims suffer from a broad range of short- and long-term physical, cognitive and emotional impairments. Mild TBI (mTBI) (the majority of TBIs) is difficult to diagnose since by using C.T. or MRI, patients fail to show any clear brain defects^1^,^2^. Nevertheless, mTBI patients frequently suffer from long-lasting cognitive, sensory and emotional deficits^3^.

B-TBI is a TBI form (originating from combat zone activities), which is defined as an injury imposed on the brain after a blast detonation of an improvised explosive device (IED), causing an increased incidence of complex TBIs^4^. These explosive devices can cause primarily a shock wave dependent damage (blast damage) to multiple organ systems, including the brain. They are becoming a significant medical problem for both patients and military and civilian healthcare providers^5^,^6^. The primary injury is caused solely by the changes in atmospheric air pressure producing compression and expansion of tissues and fluid filled regions of the brain. The consequences of these ‘lesion-less’ injuries often result in deficits in military personnel reaction times, spatial memory functions and in an increased occurrence of headaches, dizziness, sleep disturbances, anxiety and other emotional and behavioral changes^7^. Revealing the mechanisms that underlie the cognitive and psychological disorders induced by blast injuries highly requires further study. Thus, we have previously established a novel mouse model of a mild blast-TBI which resembles, as much as possible, an open air explosion. Blast-exposed mice suffered from a significant decline in their cognitive performance, while MRI screening showed no apparent changes in brain morphology. Nevertheless, T1 weighted images showed an increased BBB permeability 1-month post-blast. DTI analysis showed an increase in fractional anisotropy (FA) and a decrease in radial diffusivity all of which may represent brain axonal and myelin abnormalities^8^.

The injured adult central nervous system is an inhibitory environment for axon regeneration, resulting in poor neuronal recovery. It is partially due to a specific “axon regeneration inhibitors” (ARIs) accumulation at the injury sites. This collection of protein molecules includes MAG (myelin associating glycoprotein) NOGO and oligodendrocyte-myelin glycoprotein (OMgp)^9^,^10^,^11^,^12^. Gangliosides, sialic acid-bearing glycosphingolipids, are expressed at high abundance and complexity in the brain. Four gangliosides - GM1, GD1a, GD1b and GT1b compose 96% of brain gangliosides. An altered ganglioside expression resulted in neural disorders, including seizures and axon degeneration^13^,^14^. MAG, on the innermost wrap of the myelin sheath, binds to gangliosides GD1a and GT1b on axons with high affinity and to GM1 with moderate affinity. MAG-ganglioside binding ensures optimal axon-myelin cell-cell interactions, enhances long-term axon-myelin stability but also inhibits axon outgrowth after injury when the affinity is too high. Thus, it was reported that the GM1 treatment had a neuroprotective effect in various neuronal disorders^15^,^16^. Knowledge of the molecular metabolism of brain gangliosides may provide opportunities to enhance recovery after nerve injury. In functional studies, inhibition of axon outgrowth induced by MAG was reversed by sialidase, by the ganglioside biosynthetic inhibitor P4 (1-phenyl-2-hexadecanoylamino-3-pyrrolidino-1-propanol) and by antibodies to GD1a or GT1b^12^. Together, these data implicate gangliosides as functional MAG receptors that are responsible for axon outgrowth inhibition. It has been proposed that MAG engages and clusters GD1a or GT1b at the axon surface, halting axon outgrowth. In support of this concept, antibody-mediated cross linking of gangliosides GD1a or GT1b at the nerve cell surface mimicked MAG inhibition^10^. The above may lead to considering potential therapeutic implications of gangliosides as regulators of axon regeneration after injury.

Our working hypothesis suggests that the blast induced an alteration in the brain- ganglioside composition, mainly the reduction in GM1:GD1a content, leading to an axonal outgrowth inhibition and the subsequent cognitive damage. Hence, the main goal of the present study was to test the possible neuroprotective effect of GM1 administration on B-TBI mice, regarding the axonal regeneration, ARI’s expression and cognitive outcome.

## Experimental Procedures

**Figure 1:**
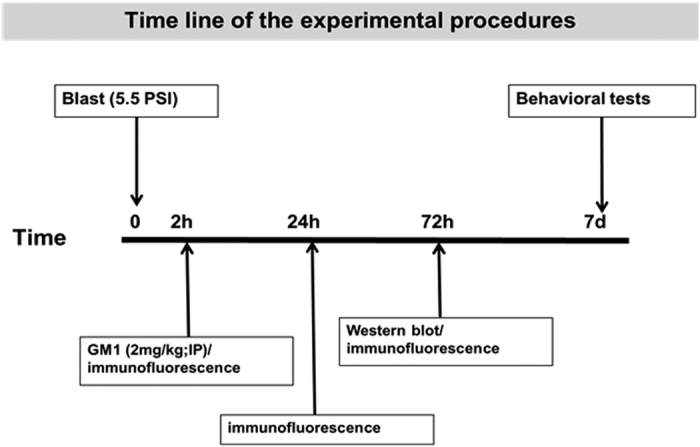
Timeline of the experimental procedures following blast-TBI. Six week old ICR mice were subjected to 5.5 PSI explosion as described in the ‘experimental procedures’. GM1 (2 mg/kg) was injected 2 hr post injury. We performed the behavioral tests 1-week post injury while for biochemistry, immunohistochemistry and ELISA mice were sacrificed 2, 24 and 72 hrs post injury as described.

*Timeline of the experimental procedures following blast-TBI is shown in Fig. 1. mice were subjected to 5.5 PSI explosion as described in the ‘experimental procedures’. GM1 (2 mg/kg) was injected 2 hr post injury. We performed the behavioral tests 1-week post injury while for biochemistry, immunohistochemistry and ELISA mice were sacrificed 2, 24 and 72 hrs post injury as described below.

### Animal studies

A series of parallel animal studies were undertaken where GM1 (2 mg/kg; IP) was administered 2 hours after a B-TBI to half of the injured mice. A separate group of naive animals were administered with GD1a (2 mg/kg; IP). Hippocampal and cortical axonal outgrowth, ganglioside expression and myelin proteins level were evaluated on day 3 after blast. Immunofluorescent images of myelin-axon interaction were taken 2, 24 and 72 h post blast. Animal cognition was evaluated over a period of 4 days starting on day 7 after blast.

Male ICR mice weighing 30–40 g were kept five per cage under a constant 12 hr light-dark cycle, at room temperature (22 ± 2 °C). Food (Purina rodent chow) and water were available *ad libitum*. Each mouse was used for one experiment and for one time-point only. The Ethics Committee of the Sackler Faculty of Medicine approved the experimental protocol (M-10-034), in compliance with the guidelines for animal experimentation of the National Institutes of Health (DHEW publication 85–23, revised, 1995). The number of animals per treatment group for immunofluorescence, biochemistry and animal cognition were selected based on experience from prior studies, and specific numbers are provided in the following. All attempts were made to minimize both the number of mice used for our studies and their suffering. All experimental manipulations were conducted during the light phase of the light-dark cycle. At the time of animal euthanasia, a two-stage method approved by the Sackler Faculty of Medicine Ethics Committee was used; animals were terminally anesthetized with isoflurane, followed by decapitation. However, for immunohistochemical analyses, animals were deeply anesthetized, using a combination of ketamine (100 mg/kg) and xylazine (10 mg/kg). They were then perfused transcardially with 10 mL of phosphate-buffered saline (PBS), followed by perfusion with 20 mL of a 4% paraformaldehyde (PFA) buffer. Brain tissues were dissected and stored appropriately until time of study by either immunohistochemistry.

#### Drug administration

Gangliosides GM1 and GD1a (Calbiochem USA) were first solubilized in DMSO or Methanol, respectively to 100 mg/kg and then in saline to the final concentration, 2 mg/kg. GM1 was injected (IP) 2 h post blast. GD1a was injected to naive mice 1 week prior to the cognitive tests. Control mice received a vehicle injection at the same time points (1% DMSO/methanol in saline).

#### Induction of blast TBI

Experimental conditions used to create a B-TBI and the subsequent model characterization have been described in detail elsewhere^8^,^17^. Briefly, mice were anaesthetized with a combination of ketamine (100 mg/kg) and xylazine (10 mg/kg). Once the animals were fully anesthetized, they were positioned on an elevated platform (raised 1 m from the ground) in a circle, 4 m from the source of the detonation. The animals were placed with their heads to the blast overpressure. The blast shockwave pressure generated by the detonation (500 g of trinitrotoluene [TNT]) was measured by pressure sensors, positioned identically to that of the animals (Free-Field ICP Blast Pressure Sensor; Model 137; PCB Piezoelectronics, Depew, NY, USA). The source of the blast was elevated 1 m from the ground; the maximum overpressure generated by the detonation was 5.5 PSI (37.9 kPa). The following animal treatment groups were used: control (no blast), blast and blast/GM1.

##### Biochemistry

Whole brains were removed at 2, 24, 72 h, and 1week post B-TBI. The cortex and hippocampus samples (right and left, separately) were immediately frozen in liquid nitrogen and homogenized with T-PER Tissue Protein Extraction Reagent (Pierce, Rockford, IL), with appropriate protease inhibitors (Halt Protease Inhibitor Cocktail; Sigma-Aldrich).

#### Western blots

Samples (85 μg/lane) were run (in triplets) on 4–20% Mini-Protean TGX gels (Bio-Rad) and transferred to nitrocellulose membranes. Blots were blocked for 1 h with Tris-buffered saline, containing 0.01% Tween-20 and 5% BSA or powdered milk. Membranes were incubated with primary antibodies against MBP and MAG (ab40390 and ab89780, respectively; diluted 1:1000, overnight at 4 °C, Abcam; Cambridge, UK) and then incubated with secondary horseradish peroxidase-linked antibodies (11-035-003 and 115-035-003, Jackson Immunoresearch, West Grove, PA) at room temperature for 1 hr. Bands were recognized by enhanced chemiluminescence (Pierce Rockford, IL) and exposed to an X-ray film. Protein band intensities were quantified by using the “ImageJ” software. Uniform loading was verified by stripping and re-probing with antibodies against tubulin (sc-5286; 1:1000; Santa Cruz Biotechnologies). Stripping was done after washing the membrane 5 times with TBST and then incubating with stripping buffer (0.76 g Tris base, 2 g SDS and 700 μl β-mercaptoethanol in 100 ml DDH_2_O) for 30 minutes at 50 °C (with slight agitation). Then, the membrane was washed 5 times for 5 minutes each in TBST.

Indirect enzyme-linked immunoassay (ELISA) test was used for GM1 ganglioside quantification and was performed according to ABCAM protocol (UK; http://www.abcam.com/ps/pdf/protocols/Indirect%20ELISA%20protocol.pdf). 96 well plates (Corning, Sigma-Aldrich) were coated with the cortex samples over night at 4 °C. After washing 3 times with PBS, 200 μL blocking buffer (5% non-fat dry milk in PBS) was added and incubated for 2 h in R.T. Rabbit anti ganglioside GM1 (ab23943 Abcam, Cambridge, UK) diluted 1:1000 (Dilutions were made in PBST and 2% normal horse serum) was applied on the wells for 2 h in R.T. Following washing (5 times) peroxidase-conjugated Goat Anti-Rabbit secondary antibody (Jackson ImmunoResearch PA/USA) was added for 90 min. HRP chromogen (TMB, R&D systems) was then added after washing. The results were calculated using a four-parameter curve fit and expressed as ng/ml, then normalized relative to the control (100%).

### Immunohistochemistry for mice brain slices

2, 24 or 72 h post B-TBI, mice (n = 4–6 for each group) were anesthetized with a combination of ketamine (100 mg/kg) and xylazine (10 mg/kg) and perfused transcardially with PBS and then with 4% paraformaldehyde (PFA) in 0.1 M phosphate buffer, pH 7.4. Their brains were removed, fixed overnight in 4% PFA in 0.1 M phosphate buffer, pH 7.4, and then placed in 30% sucrose for 48 h. Frozen coronal sections (30 μm) were then cut on a sliding microtome and collected serially. The free-floating sections were first blocked by incubation with 0.1% Triton X-100 in phosphate-buffered saline and 10% normal horse serum for 1 h at 25 °C. Primary antibodies (mouse anti Pan axonal neurofilament marker SMI 312 and mouse anti neurofilaments, phosphorylated, SM1-31R Covance; USA, Diluted 1:1000 in incubation buffer; mouse anti GD1a ganglioside MAB5608, EMD Millipore, Diluted 1:1000 in incubation buffer; rabbit anti myelin basic protein, rabbit anti ganglioside GM1 and rabbit anti F-actin (ab40390, ab23943, ab205, respectively; Abcam, Cambridge, UK) diluted in PBST and 2% normal horse serum, and incubated with the sections for 48 h at 4 °C. Control slices were stained by omitting primary antibodies. After being rinsed in PBST the sections were incubated for 1 h at 25 °C with DyLight^TM^594-conjugated AffinityPure Donkey Anti-Rabbit IgG and DyLight^TM^488-conjugated AffinityPure Donkey Anti-mouse IgG (diluted 1: 300; ab150076 and ab150109, respectively; Jackson Laboratories, Bar Harbor, ME, USA). After rinses in PBST, free-floating sections were mounted on dry gelatin-coated slides, and fluorescence was visualized using a Zeiss LSM 510 confocal microscope (Carl Zeiss, Jena, Germany) or Leica SP5 confocal microscope (Leica, Germany). Excitation light was provided by the 488 nm line of argon lasers for the DyLight-488 fluorophore, the 543 nm line of HeNe lasers for the DyLight-594 fluorophore and 405 excitations for DAPI. All the fluorescence images for a certain figure were taken with the same scanning parameters. The quantification of GM1, GD1a and pNFH expression and the co-localization analysis were done using the Imaris software, (Bitplane AG, Zurich, Switzerland). Overall, we had 2–4 sections (different bregmas) from each brain. Images were taken from 3 fields (from each section) at the post central cortex and the hippocampus. The images from each section and from each animal were averaged for the final value.

#### Cognitive assessments

The Novel object recognition test. An object recognition test was used to evaluate recognition memory^18^. This task is based on the tendency of rodents to discriminate between a familiar object and a new object. The open field was a 59 × 59 cm arena, surrounded by 20 cm black plexiglass walls. The floor of the arena was also black and divided into 36 identical squares by white gridlines. Each mouse was placed in an empty arena for a 5 min habituation. After 24 h, the mice were placed for 5 min into the arena with two identical objects, A and B (e.g., bottles), positioned 40 cm from each other and 10 cm from the walls. On the next day (day 3), the mice were placed once again for 5 min into the arena with object A (the same as on the second day) and object C (a new object; e.g., a coffee can). The arena and the objects were cleaned with 70% ethanol between each trial. Exploration of an object was defined as rearing on the object or sniffing it at a distance of less than 2 cm and/or touching it with the nose. Discrimination of recognition novelty was assessed by a preference index^19^: (time exploring the new object − time exploring the old object)/(total time exploring an object). Mice that spent less than 10% of the total time (30 sec) near the objects were excluded from the analysis.

Y-maze test. Spatial memory was assessed by using the Y-maze, which was first described^20^ and then subsequently validated as a task -requiring hippocampal function and spatial memory^21^. The Y-maze was constructed of black Plexiglas with three identical arms (30 × 8 × 15 cm). Overt cues were attached inside of the Y-maze. The test included two trials separated by a two-minute interval. The first trial was 5 minutes with only two arms open (the start arm and the arm called “the old” arm), and the third arm was blocked by a door (the novel arm). The mouse was put in the start arm in the part most distant from the other two arms. After the first (familiarization) run the mouse was put back into the cage for two minutes. The second run lasted two minutes, and all three arms were open. Time spent in each of the arms was measured. After each run and after each mouse the maze was cleaned with 70% ethanol. The new arm preference index was calculated as follows: (time in the new arm − time in the old arm)/(time in the new arm + time in the old arm).

Mice that spent less than 10% of the total time (30 sec) in the arms were excluded from the analysis.

### Data analysis

All results are given as mean ± SEM and data were analyzed using one-way ANOVA; statistical significance was set at p < 0.05. P values of post hoc tests were adjusted using the Tukey HSD test and a nominal significance level of 0.05 was used.

## Results

### Blast injury significantly reduced the expression of GM1 ganglioside in the brains of the injured mice

**Figure 2:**
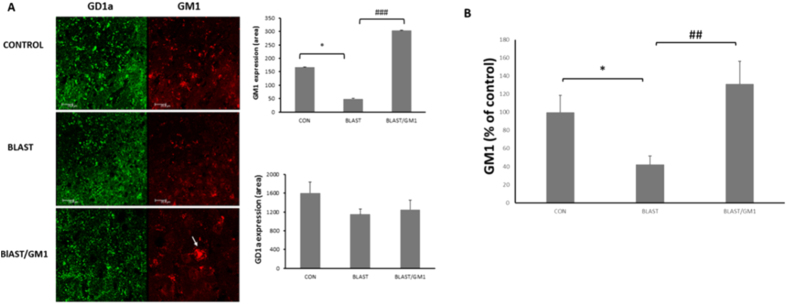
B-TBI significantly reduced the expression of GM1 in the cortex. (**A**) The expression of GM1 (red) in the cortices of blast exposed mice was significantly reduced compared with control mice 72 h post injury. GM1 administration (2 mg/kg; IP) not only prevented this reduction but also reorganized this ganglioside in big clusters. F(2, 15) = 17.7; p < 0.0001; n = 4–6). Neither blast nor GM1 treatment had a significant effect on GD1a expression (green). (**B**) Indirect enzyme-linked immunoassay (ELISA) test also revealed a significant reduction in GM1 content in the cortex of the injured mice compared with control, which was significantly prevented by the administration of GM1. F(2, 13) = 5.31; p < 0.05.

Our basic hypothesis refers to the alteration in ganglioside expression as one of the mechanisms that underlie the damage in the brains of blast injured mice (AOI). Hence, we evaluated the expression of GM1 and GD1a gangliosides by immunofluorescence staining, using specific antibodies raised against these molecules. Figure 2A shows that 72 h post injury, GM1 expression was significantly reduced in blast brains compared with control (166.3 ± 33.2 and 49.2 ± 8.2, respectively; *p < 0.05; n = 6 for every group). GM1 administration (2 mg/kg; ip) not only prevented this decline but also induced a significant, pronounced increase in GM1 signal intensity in the cortex (303.7 ± 39.7; ^**###**^p < 0.001). Moreover, GM1 was re-organized in large clusters in the cortex of blast/GM1 mice. Neither blast nor GM1 treatment had any effect on GD1a expression (Fig. 2A). In addition, an Indirect enzyme-linked immunoassay (ELISA) test also revealed a significant reduction in GM1 content in the cortex of the injured mice compared with control mice (20.28.3 ± 4.5, n = 5; and 47.88 ± 8.9, n = 6, respectively; *p < 0.05). GM1 treatment to blast mice prevented this decline significantly (62.74 ± 11.9; ^**##**^p < 0.01; n = 5). The data were normalized relatively to 100% in the control mice (Fig. 2B).

### Blast-TBI significantly elevated axon-myelin interaction

**Figure 3:**
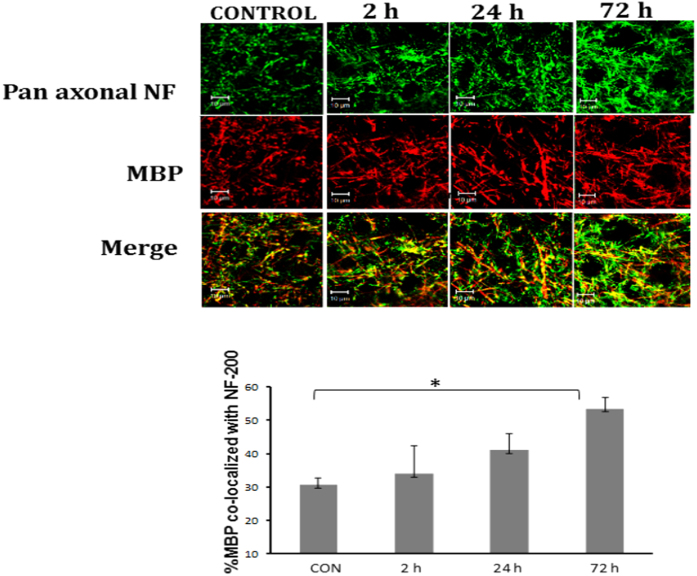
A time–dependent increase in axon-myelin interaction after blast. Axon-myelin interaction was evaluated after double-staining brain slices of mice with antibodies raised against MBP (red, for myelin) and pan axonal neurofilaments marker (green, for axons). The level of yellow color (merged) represents the interaction. It was gradually elevated following blast and reached significance at 72 h post injury. [F(3, 8) = 3.73; p = 0.0001].

Our working hypothesis suggests that the elevation in GD1a/GM1 ratio might shift the interaction of the myelin proteins towards GD1a and to an elevated affinity of the myelin-axon interaction. Our next test was aimed at evaluating the interaction of these 2 components using immunofluorescence staining and analysis. In order to do so we double-stained brain slices of mice with antibodies raised against MBP (red, for myelin) and pan axonal neurofilaments marker (green, for axons). The amount of yellow color (merged) represents the interaction. Figure 3 shows that the interaction was gradually elevated following blast and reached significance at 72 h post injury (Fig. 2). The values of intensity increased from 30.67 ± 1.9, n = 5, to 53.53 ± 3.34, n = 3; *p < 0.05.

### GM1 administration after the injury prevented blast-induced axon-myelin interaction

**Figure 4:**
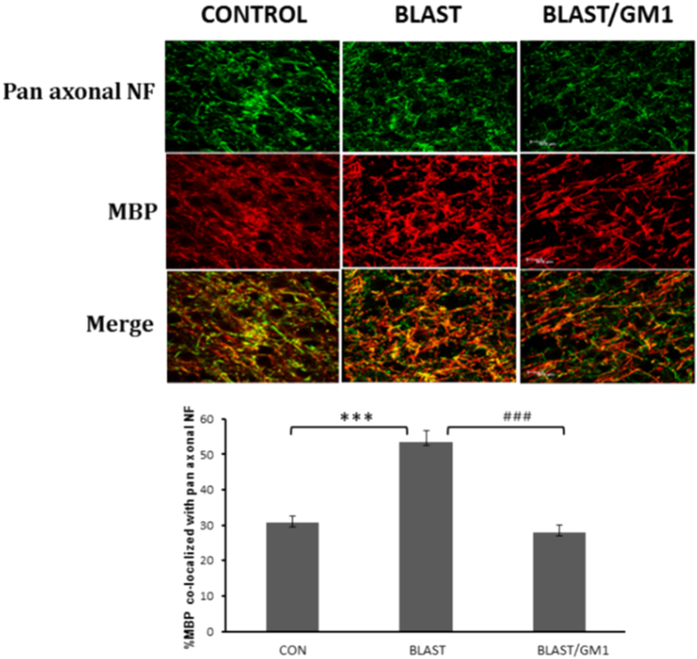
GM1 administration to blast-injured mice prevented the elevation in axon-myelin interaction. GM1 administration (2 mg/kg ip) completely prevented the elevation in the interaction between MBP (red) and axonal neurofilaments (green) (72 h post blast, representing axon-myelin interaction, yellow). [F(2, 13) = 39; p = 0.0001].

In order to establish our hypothesis, we tested the effect of GM1 administration on the blast-induced myelin-axon interaction (MBP in red for the myelin; pan axonal neurofilaments in green; co-localization in yellow for interaction). Figure 4 shows that GM1 injection completely prevented the elevation in myelin-axon co-localization (53.5 ± 3.34, n = 9, in blast brain compared with 28.01 ± 1.9, n = 9 in blast/GM1 brains; ^#^p < 0.001; n = 10; Fig. 4).

### Blast injury induced a significant decline in the axonal marker pNF-H in the cortex and in the hippocampus

**Figure 5:**
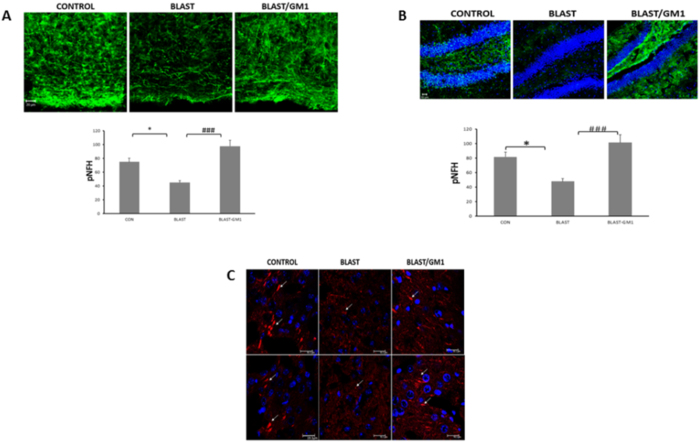
The neuroprotective effect of GM1 administration on axonal and growth cones damage as seen by phosphorylated NF-H and F-actin expression. Phosphorylated NF-H (green) positive neurons in the cortex (**A**) and hippocampus (**B**) [F_(2,12)_ = 14.134, *p* = 0.0007]; [F_(2,12)_ = 5.69, *p* = 0.018]. Values are mean ± SEM, of n = 4–6 mouse brains. (**C**) Representative images of cortex slices immuno-stained with F-actin antibodies which identify growth cones. Blast injury induced shrinking/collapse of the growth cones. GM1 treatment inhibited this effect. (the white arrows point to growth cones).

Since we suggest AOI (axonal outgrowth inhibition) as the result of GM1 decline, the next experiments were aimed at evaluating the effect of GM1 on the expression of the phosphorylated NF-H (the axonal heavy neurofilaments) as seen in immunofluorescence microscopy. As expected in our model, blast injury significantly reduced the expression of pNF-H in the cortex: 74.8 ± 5.13, n = 5 in control brains compared with 44.75 ± 2.59, n = 4 in blast mice (*p < 0.05). GM1 prevented this decline (97.5 ± 8.6; ^#^p < 0.001; n = 6; Fig. 5A). Similarly, GM1 blocked the reduction of pNF-H in the hippocampi of blast injured mice. The signal intensity declined from 81.61 ± 6.6, n = 6, to 47.62 ± 4.01, n = 7; *p < 0.05. GM1 prevented this decline: 101.24 ± 10.95, n = 6; ^###^p < 0.0001; Fig. 5B). Figure 5C demonstrates the effect of blast-injury on the growth cones in the cortex. The white arrows point to representative growth cones (visualized by immunostaining with anti F-actin antibodies). One can clearly see that the growth cones in the control brains are much bigger with the classic “conus shape”. In the blast brains, the staining revealed a different, smaller size and organization. In GM1-treated brains, the typical growth cones appeared again.

### The effect of GM1 treatment on the expression of the myelin proteins in the blast injured brains

**Figure 6:**
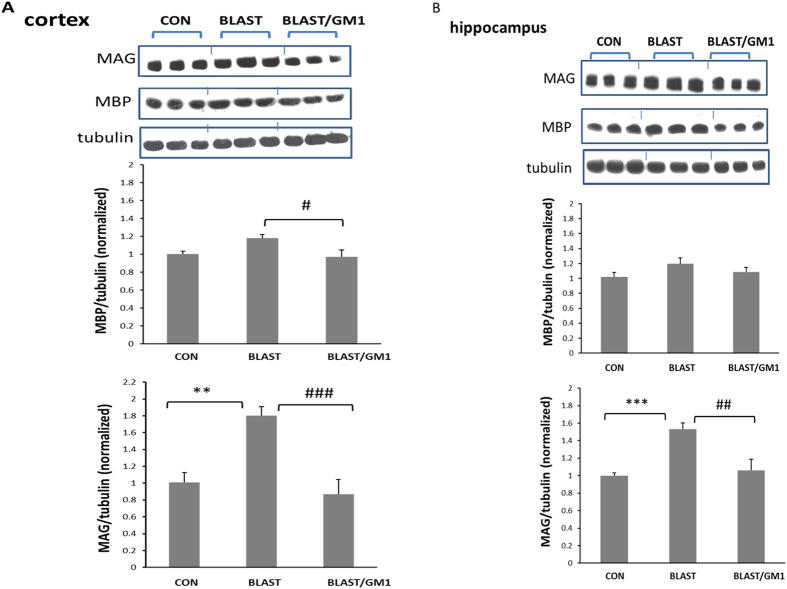
GM1 administration prevented the elevation in MAG expression in the brains of B-TB1 mice. Cortex (**A**) and hippocampus (**B**) protein extracts (separate samples) prepared from sham or B-TBI animals at 72 h post injury were subjected to gel electrophoresis, followed by immunoblot analysis using antibodies against MAG or MBP. The expression level was evaluated by densitometry analysis. One-way ANOVA revealed a significant elevation in MAG expression following blast (Tukey *post hoc* test). This elevation was completely blocked by GM1 treatment (2 mg/kg;ip). Cortex: [F(2, 24) = 13.4; p = 0.0001]; hippocampus: [F_(2,24)_ = 11.14, *p* = 0.001). MBP (myelin basic protein), on the other hand, was not altered by the blast or by GM1 treatment.

It was previously suggested that ARI’s (axonal regenerating inhibitors) originating from myelin are also responsible for axonal damage. Thus we evaluated the level of the myelin proteins by western blot analysis. As seen in Fig. 6A, blast injury significantly elevated the inhibitory protein MAG (myelin associated glycoprotein) in the cortex: 1.8 ± 0.10 compared with control 1.0 ± 0.10; **p < 0.01. This elevation was prevented by the administration of GM1 2 h post blast: 0.87 ± 0.17; ^###^p < 0.001; n = 9 for all groups. On the other hand, blast did not change MBP (myelin basic protein): 1.00 ± 0.03 and 1.17 ± 0.04 (control and blast, respectively; n.s; n = 6 for all groups; Fig. 5A). Similar results were obtained in the hippocampus: MAG was significantly elevated (1.00 ± 0.03 in control compared with 1.53 ± 0.06 in blast; n = 9 for all groups; *p < 0.001). GM1 significantly prevented this effect (1.06 ± 0.13; ^#^p < 0.01;). As in the cortex, blast did not change the level of MBP (1.00 ± 0.06, 1.19 ± 0.07 and 1.09 ± 0.06 in control, blast and blast/GM1 brains, respectively; n = 7 for all groups (Fig. 6B). These results show that the blast induced an elevation in MAG expression but not in MBP.

### GM1 administration ameliorate the significant damage to the cognitive outcome after B-TBI

**Figure 7:**
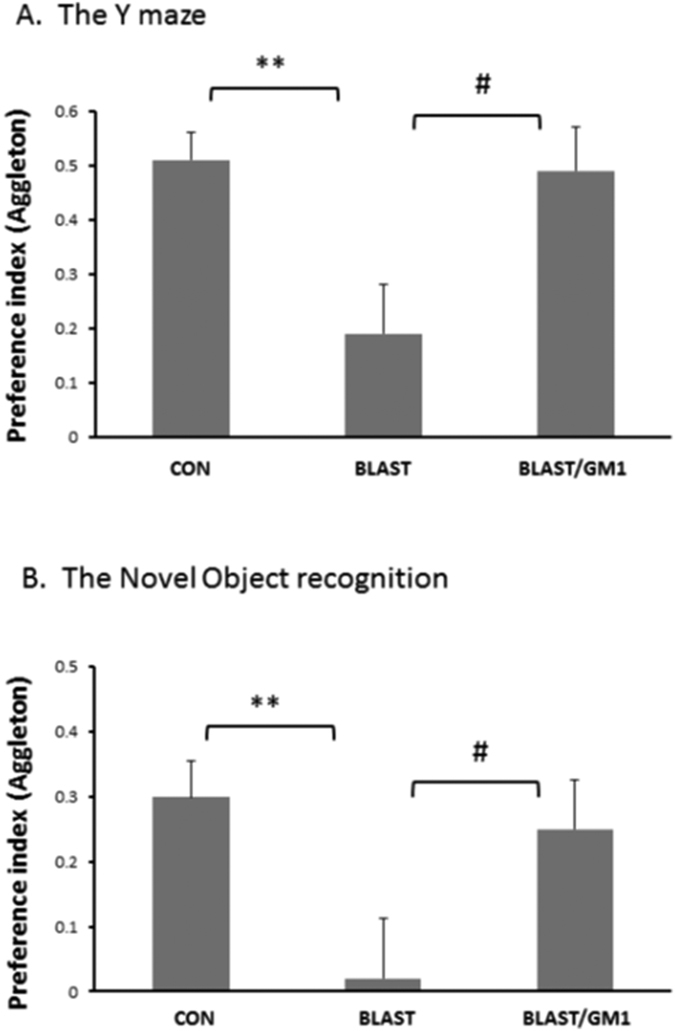
The neuroprotective effect of GM1 administration on the cognitive performance after blast-TBI. The behavioral tests were performed 7 days post B-TBI (separate groups). (**A**) The recognition memory was assessed by the Novel Object Recognition test (OR) by calculating the relative time that the mice spent near a novel object compared to an old, familiar one (“preference index”, see methods). The significant decrease of preference index in mTBI mice was significantly prevented by the administration of GM1 (2 mg/kg;IP). One way ANOVA revealed significant effect of group: [F(2, 20) = 6.28;p = 0.0007]; [F(2, 35) = 8.78; p = 0.0008] (Tukey *post hoc* test).

The last set of experiments was aimed at testing the hypothesis that alteration in the composition of gangliosides in the brain following B-TBI may be responsible for the cognitive deficits. Indeed, a single dose of GM1 (2 mg/kg, IP) administered to mice after B-TBI prevented the blast-induced cognitive deficits. As expected, GM1 prevented the spatial memory deficits after B-TBI as was tested by the Y-maze. Seven days post blast the preference index was significantly lower in B-TBI mice compared with control (0.51 ± 0.05, n = 14 and 0.19 ± 0.09, n = 14, respectively; **p < 0.01). This impairment was significantly prevented by GM1 (0.49 ± 0.08; ^**#**^p < 0.05; n = 12; Fig. 7A). Similarly, B-TBI reduced the visual/recognition memory as seen by the Novel Object recognition test 7 days post injury. The preference index declined from 0.30 ± 0.05, n = 8, to 0.02 ± 0.09, n = 7; *p < 0.0). GM1 administration to B-TBI mice abolished this decline in the preference index (0.25 ± 0.07; ^**#**^p < 0.05; n = 6; Fig. 7B).

### GD1b injection to naive mice induced cognitive deficits, which were prevented by the administration of GM1

**Figure 8:**
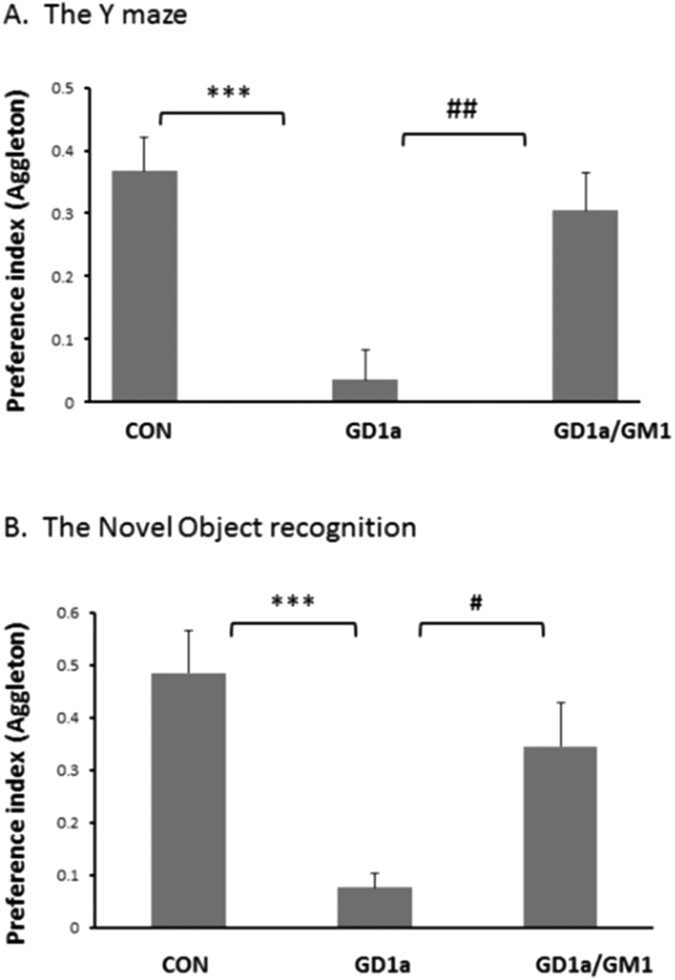
The neuroprotective effect of GM1 administration on the cognitive performance after GD1b injection. GD1b administration (1 mg/kg; IP) induced a similar cognitive damage as blast did. (**A**) The recognition memory was assessed by the Novel Object Recognition test (OR) by calculating the relative time that the mice spent near a novel object compared to an old, familiar one (“preference index”, see methods). The significant decrease of preference index in GD1b mice was significantly prevented by the administration of GM1 (2 mg/kg;IP). One way ANOVA revealed significant effect of group: [F(2, 26) = 9.00; p = 0.0003]; [F(2, 25) = 11.3; p = 0.0003] (Tukey *post hoc* test).

We hypothesized that the blast altered the composition of gangliosides, leading to a reduction in the level of GM1 and a possible elevation in GD1b gangliosides in the brain, which leads to axonal outgrowth inhibition due to elevated affinity of the myelin inhibitory molecules to GD1b and thus, cognitive damage. The last set of experiments was performed in order to mimic this effect of blast on the ganglioside composition by injecting GD1b to naive mice. Indeed, GD1b (2 mg/kg, IP) significantly damaged spatial memory as seen in the Y maze test: preference index declined from 0.37 ± 0.05, n = 10, to 0.036 ± 0.04, n = 9; ***p < 0.001. GM1 treatment prevented this damage: 0.31 ± 0.06, n = 8; ^##^p < 0.01. A similar damage was found in the visual memory performance as seen in the NOR: the preference index in blast mice was reduced from 0.48 ± 0.08, n = 12, to 0.07 ± 0.03, n = 9, (*p < 0.05). As seen in Fig. 8, GM1 administration prevented this decline (0.34 ± 0.08, n = 8; ^#^p < 0.05; n = 9–12).

## Discussion

It was previously suggested that an alteration in the composition of gangliosides in the CNS might lead to AOI (axonal outgrowth inhibition) by elevating the interaction of myelin with GD1a and GT1b on the axonal surface and hence, reducing the level of axonal regeneration^12^,^22^,^23^. In addition, other published studies reported the inhibitory effect of the myelin (mainly NOGO and MAG) on axonal regeneration^24^,^25^,^26^,^27^. These studies were done mainly on spinal cord injury. The present study is testing this theory in a blast-TBI model in the brain. We suggest that blast may affect the composition of gangliosides in the brain, leading to the cognitive and behavioral deficits at least partially due to the axonal outgrowth inhibition (AOI). The present study is aimed at characterizing this route by measuring the ganglioside composition and the inhibitory myelin protein (MAG) expression in the brains of mice subjected to blast injury. We used GM1 administration (2 mg/kg; IP) not only for evaluating a possible treatment but also for testing our working hypothesis. Indeed, even one low dose of GM1 (IP) injection was sufficient to show neuroprotection following a blast-TBI in mice, both in regard to the axonal damage and cognitive impairments.

It was essential to reveal this pathway, beginning with the role and expression of the main gangliosides, GM1 and GD1a after blast. Our hypothesis pointed to 3 possible results: reduction in GM1 expression, elevation in GD1a expression, or both, all of which present a reduction in GM1/GD1a ratio. Figure 2A shows that the explosion significantly reduced the expression of GM1 in the cortices of mice. Moreover, GM1 treated mice, not only exhibited a significant elevation in GM1 expression, but also this ganglioside was re-organized in big clusters as was found previously^28^,^29^. Our measures did not identify a change in GD1a expression, nevertheless, the fact that GM1a was significantly reduced in blast brains and elevated dramatically in GM1 treated mice support our hypothesis regarding GM1/GD1a ratio. Moreover, similar results regarding the effect of blast on GM1 content in the cortices of the injured mice and the prevention of this by GM1 treatment were obtained from an indirect enzyme-linked immunoassay (ELISA) test (Fig. 2B). The effect of blast on the ganglioside expression, together with the effect of GM1 administration to blast mice, strongly supports our working hypothesis regarding the involvement of these gangliosides in blast-induced damage.

Since our working hypothesis is AOI (axonal outgrowth inhibition), it was important to test the effect of blast on newly synthesized neurites or axons. As we were working with tissue sections and not isolated neurons, we couldn’t measure the actual length. We measured the signal intensity of the expression of the phosphorylated heavy neuro-filaments (pNF-H) in both the cortex and hippocampus (Fig. 5A,B). pNF-H are localized mainly in axons, especially in the projections, building cross-bridges^30^,^31^,^32^. Moreover, it was previously suggested to be involved in axonal outgrowth^33^. In animal models with reduced expression of pNF-H, neuronal damage and pathological conditions were found leading to neurodegeneration (for review see ref. 31). Thus, evaluating the expression of pNF-H could imply for neuronal damage and recovery. Indeed, Fig. 5A,B show that the blast induced a significant decline of pNF-H in the cortex as well as in the hippocampus. Growth cones are dynamic, actin-supported extensions of a developing neurites and as such may serve as markers for axonal regeneration^34^. F-actin stained brain sections revealed that the blast injury reduced the size and changed the shape of the growth cones in the cortices of the blast-injured mice (Fig. 5C). This may suggest a blast-induced growth-cone collapse. These results regarding the effect of blast on both pNF-H and F-actin expression further support our hypothesis that it reduced axonal regeneration. GM1 treatment 2 h post injury prevented pNF-H reduction, and inhibited the alteration in the shape and size of the growth cones, implying for the involvement of GM1 levels in axonal regeneration.

An essential step for supporting the AOI theory was the involvement of the inhibitory myelin associated glycoprotein (MAG). It was previously reported to take part in the inhibitory effect of the myelin on axon regeneration^9^,^25^,^26^,^35^. The fact that MAG was significantly elevated in blast brains (and that myelin basic protein MBP was not) supported the hypothesis that it played a role in the AOI. In addition, it showed that blast elevated MAG expression and not just activated it. Moreover, GM1 treatment completely prevented this elevation, endorsing our basic theory, regarding the interaction of MAG with the GD1b ganglioside on the axon surface. It also revealed that MAG expression was negatively regulated by GM1. Another inhibitory myelin protein, NOGO was reported in many studies to play a cardinal role in the inhibitory effect of myelin^24^,^36^,^37^. Suppressing its expression in neural cultures as well as in animal models prevented the axonal outgrowth inhibition^38^,^39^,^40^,^41^. Preliminary results of NOGO expression by immunofluorescence staining showed an elevation in the cortices of the injured brains and that GM1 administration prevented this elevation (data not shown).

MBP (myelin basic protein) is not an inhibitory molecule, and is involved in myelin stability and compaction^42^,^43^. The fact that the inhibitory MAG (and maybe NOGO) were elevated in the blast-injured brains but not MBP, suggest that this is a specific mechanism that involves only the ARIs and not the other components of the myelin. Since GM1 prevented the elevation of MAG, it strongly suggests the co-involvement of gangliosides and ARIs in the destructive mechanisms following blast injury.

AOI theory suggests that due to an elevation in GD1b/GM1ratio, there is an elevated interaction with MAG, leading to a tighter interaction between the axon and the myelin sheath (which inhibits the axonal regeneration ability)^10^,^35^,^42^,^43^. We show in Figs 3 and 4 that when double staining brain slices with antibodies raised against pan axonal neurofilaments (green) and against MBP (red), the yellow color may represent the interaction of the two. Indeed, one can clearly see that this interaction is significantly elevated at 72 h post blast injury. Moreover, GM1 treatment prevented this phenomenon. Thus, this study implies that the exposure to blast might induce significant axonal injury followed by cognitive deficits partially due to AOI (axonal outgrowth inhibition). We showed that a change in the ganglioside composition and MAG expression were involved in this process. Moreover, the results also imply that GM1 regulates in some way the expression of MAG, since when we prevented the decline of GM1 we also prevented the elevation of MAG.

GM1 significantly prevented the damage to the visual/executive as well as to the spatial memories (as seen by the novel object recognition and the Y maze tests, respectively; Fig. 6). This implied that the gangliosides were involved in the mechanisms that underlie the blast-induced brain damage in our model.

In order to test the main theory regarding the hypothesis that the reduction in the relative expression of GM1 compared with GD1b induced the damage, we mimicked this effect by injecting exogenous GD1b to naive mice. This had a dramatic effect on the cognitive performance, similar to the effect of blast, which was prevented by GM1. These results further imply that GM1/GD1b ratio had some role in the destructive pathway after the exposure to the explosion.

Our results may suggest GM1 as an optional pharmacological strategy for blast-induced TBI and perhaps for other brain injuries. GM1 was suggested previously as a neuroprotective treatment in some neurological disorders mainly in neurodegenerative diseases. It was used in much higher doses and in a continuous administration^15^,^16^. The fact that in our model a single low dose of GM1 (2 mg/kg) was sufficient for neuroprotection may be of importance. It could be that treating with GM1 shortly after the blast, will prevent the cascade of the events that lead to neurodegeneration, yet more studies are needed for both find a therapeutic window (by a dose and time post-injury responses) and in order to learn more about the mechanisms underlying these important effects.

## Additional Information

**How to cite this article**: Rubovitch, V. *et al*. Restoring GM1 ganglioside expression ameliorates axonal outgrowth inhibition and cognitive impairments induced by blast traumatic brain injury. *Sci. Rep.* **7**, 41269; doi: 10.1038/srep41269 (2017).

**Publisher's note:** Springer Nature remains neutral with regard to jurisdictional claims in published maps and institutional affiliations.
